# Dataset on cognitive distortions for text classification in Indonesian language

**DOI:** 10.1016/j.dib.2025.111836

**Published:** 2025-06-27

**Authors:** Nyoman Putra Sastra, Linawati, Gede Sukadarmika, I.P.G.H. Suputra, Ni Made Ariwilani, Ni Luh Indah Desira Swandi

**Affiliations:** a Department of Doctoral Engineering, Faculty of Engineering, University of Udayana, Denpasar 80113, Indonesia; b Department of Informatics, Faculty of Math and Natural Science, University of Udayana, Badung 80361, Indonesia; c Department of Psychology, Faculty of Medicine, University of Udayana, Denpasar 80113, Indonesia

**Keywords:** Cognitive distortion, Sentences classification, Mental health, Natural language processing, Distorted sentences

## Abstract

The collected dataset is a novel collection of Indonesian-language sentences annotated for cognitive distortions. Cognitive distortion is generally a systematic bias in information processing that reinforce negative thinking and contribute to depression. The data was collected via a questionnaire administered to Indonesian participants aged 18 and above, capturing demographic information (anonymized), history of engagement with mental health professionals, and responses to 10 open-ended life-related questions. Two licensed psychologists manually annotated each sentence, first identifying the presence of cognitive distortions and then categorizing them into one of 10 predefined distortion types. The dataset comprises 4,662 labeled sentences, including 2,246 non-distorted and 2,416 distorted instances. To address the relatively small number of samples in certain distortion classes, data augmentation was performed using the back-translation method. This process resulted in a final dataset size of 4,992 entries. To our knowledge, this is the first Indonesian text classification dataset in the mental health domain, specifically targeting cognitive distortions. This resource is valuable for natural language processing (NLP) research, particularly in text classification tasks, and may also support computational psychology studies. The dataset provides a foundation for developing NLP tools to detect cognitive distortions in low-resource languages and contributes to mental health research in Indonesia.

Specifications Table

**Table:** 

Subject	Computer Sciences
Specific subject area	Natural Language Processing, Text Classification in the Mental Health Domain in Indonesian.
Type of data	Table, CSV dataset. Raw, Analyzed, Filtered, Processed.
Data collection	Data were obtained from a questionnaire distributed online via Google Forms. The questionnaire was compiled based on the results of discussions with psychology experts. Ten types of basic questions are directed to obtain distorted sentence responses from each respondent. There were 593 respondents in the process. Sentence data collected from each respondent were read, analyzed, and annotated to obtain a distortion label. Sentences labeled as distorted were explored in more depth and labeled according to the distortion class.
Data source location	Udayana University, Denpasar, Indonesia.
Data accessibility	Repository name: Mendeley Data Data identification number: 10.17632/k84bkv8dkt.4 Direct URL to data: https://data.mendeley.com/datasets/k84bkv8dkt/4
Related research article	None.
	

## 1 Value of the Data

• This is the first public cognitive distortion sentence dataset in Indonesian [1]. The dataset is primary data collected directly from human thought expressions based on questions compiled by psychologists, not generated by machines or robots. Table 1 presents a comparison of cognitive distortion datasets summarized from 2017 to 2025.

**Table 1:** Cognitive distortion dataset comparison.

Year	Dataset source	Language	Number of Distortion Class	Size
2017 [2]	Personal Blog (Tumblr API)	English	1 distortion class, detection task (binary)	459
2017 [3]	Daily mood log and narration of the cognitive distortion patients	English	10 class	N/A
2018 [4]	Post from Koko platform (peer-to-peer therapy)	English	15 class	4,035
2020 [5]	- Crowdsourced distortion recollections (CrowdDist) from MTurk platform - Mental health therapy logs (MH) from TAO connect platform: MH-C & MH-D	English	CrowdDist: 15 class MH-C: 15 class MH-D: binary	CrowdDist: 7,666 MH-C: 1,164 MH-D: 1,799
2021 [6]	Kaggle Dataset from Therapist Q&A	English	10 class	2,530
2021 [7]	Journaling text from Twitter API, Web Crawling, Survey, and Happy DB	English	2 class	2,409
2022 [8,9]	Real data from Clinician-client SMS dialogues	English	6 class	7,354
2022 [10]	Tweets from Twitter	Arab	5 class	9,250
2023 [11]	Social media post	English	11 class	3,644
2023 [12]	multi-modal clinical conversation transcript from doctor-patient interactions	English	N/A	N/A
2024 [13]	Generated from Chinese psychological Q&A dataset (PsyQA)	Chinese	10 class	4,001
2025 [14]	free-form text written by Korean adolescents	Korean	10 class	108,717
2025 [1]	Questionnaire through Google form platform	Indonesian	10 class	4,992
• NLP researchers can use this dataset as a reference in building automatic cognitive distortion detection and classification models. Detection for two classes, distortion, and non-distortion. Classification task is carried out different types or classes of existing distortion. This dataset can reference a training model that can be an automatic machine that can detects distortion from many users. Apart from that, for psychology scientists, this dataset can be a reference in learning about the character of Indonesian people in expressing their thoughts and what types of distortion tend to be more dominant in the generation being studied, etc.
• Researchers can use this dataset as a reference freely because, so far, many existing cognitive distortion datasets are private because they are related to privacy. The acquisition process of this dataset has received ethical clearance from the Ethical Committee on Social Studies and Humanities- National Research and Innovation Agency of the Republic of Indonesia.
• The dataset has been analyzed and validated by two annotators separately and consists of 10 complete distortion-type classes according to the theory [15]. Based on Beck (1979)[16] and Burns (1999) [15] theories, cognitive distortions can be elaborated into 10 to 15 categories. In constructing this dataset, we chose to use 10 classes because several of the 15 categories are redundant or overlapping, such as Blame and Personalization, Jumping to Conclusions consist of Mind Reading and Fortune Telling, as well as Catastrophizing also merged into Magnification or Minimization. The merging was done to simplify the annotation schema and improve inter-annotator consistency [13]. We also included markers in each sentence that contains cognitive distortions to highlight the relevant text segments. These markers were used to support the labeling process, facilitate model training, and enable more detailed analysis of the specific parts of the sentences that reflect distortions.

## 2 Background

As depression continues to pose a major global health challenge, attention to supportive mental health strategies becomes increasingly important. Among these strategies is the identification of cognitive distortions—patterns of biased thinking reflected in language that may contribute to negative affect. Advances in artificial intelligence (AI) have enabled computational modeling of mental health conditions, particularly in detecting and classifying cognitive distortions from textual or spoken data. Prior studies (2017–2025) [[2], [3], [4], [5], [6], [7], [8], [9], [10], [11], [12], [13], [14]] have explored this task across languages, employing diverse datasets and methods. However, as highlighted in [17], progress remains constrained by the scarcity of publicly available datasets, which are critical for training and benchmarking models. To address this gap, we present the first Indonesian-language dataset annotated for cognitive distortions. Indonesian was selected due to the absence of existing resources in this linguistically and culturally underrepresented context, despite the language’s widespread use by over 200 million speakers. This dataset supports the development of NLP tools for mental health applications in low-resource settings and facilitates cross-cultural research on cognitive distortions.

## 3 Data Description

This dataset was acquired from a total of 593 respondents who filled out the questionnaire via the Google Form platform. The questionnaire results showed that of the 593 respondents, around 50.8% were filled in by women, and the rest were men. Around 7.9% of 47 respondents stated that they had interacted with or visited a psychologist, but only 48% of them said that there had been a diagnosis from the psychologist they visited. The rest indicated that there was no diagnosis or that there were other needs. From this, interacting with a psychologist is something that may be considered unusual in people's lives, especially in Bali-Indonesia. It can be seen that less than 8% of respondents have ever interacted with a psychologist. Table 2 shows additional details from the questionnaire respondents' data.

**Table 2:** Additional information details of respondents.

Variable		N	Percent
Sex/gender	Male Female	292 301	49.2 50.8
Age (year)	18 19 20 21 22 23 24 25 30	226 148 111 71 21 9 1 2 1	38.31 25.08 18.81 12.03 3.56 1.53 0.17 0.34 0.17
Last education	High School Bachelor	521 72	87.86 12.14
visited a Psychologist?	Yes No	47 546	7.9 92.1
Received specific diagnosis from a Psychologist? (47 respondents)	Yes No	22 25	46.8 53.2

**Fig. 1:**
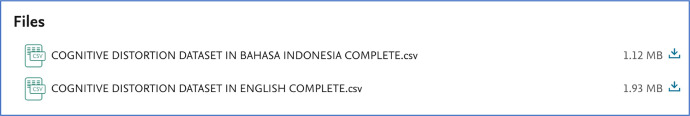
Two files (.csv) dataset in Indonesian & English.

**Fig. 2:**
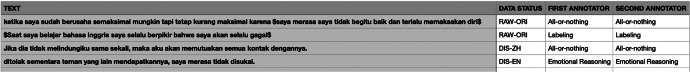
Example of dataset (.csv) open with numbers application.

The questionnaire yielded approximately 5,930 responses to its main questions, though not all responses were suitable for inclusion in the final dataset. Responses containing fewer than three words, single characters, or symbolic answers were excluded during preprocessing as they were deemed insufficient for meaningful analysis. After this filtering process, the dataset consisted of 4,662 sentences that were carefully annotated by trained professionals. Within the annotated dataset, 2,246 sentences were classified as non-distorted while 2,416 contained identifiable cognitive distortions. In the final annotation stage, we identified several classes with relatively small sample sizes. To address the relatively small number of samples in certain distortion classes, data augmentation was performed using the back-translation method [18]. We performed data augmentation using back-translation from Indonesian into Chinese (ZH), English (EN), Javanese (JV), Malay (MS), and Tagalog (TG). This augmentation was specifically applied to classes with fewer than 200 samples, namely: Mental Filter, All-or-Nothing Thinking, Magnification or Minimization, and Emotional Reasoning. The threshold of 200 samples was determined to reduce the disparity between the number of distorted and non-distorted sentences, aiming for a more balanced dataset composition. Fig. 1 shows two datasets: the file titled “COGNITIVE DISTORTION DATASET IN BAHASA INDONESIA COMPLETE.csv” contains entries in Indonesian, while the file “COGNITIVE DISTORTION DATASET IN ENGLISH COMPLETE.csv” contains the translated English versions of the same entries. Fig. 2 provides sample entries from the dataset. Further details are provided in the following explanation.

A. The “**TEXT**” column displays the original responses provided by participants, which may consist of multiple sentences. During the annotation process, segments identified as cognitive distortions were marked with surrounding “$” symbols to clearly indicate the relevant text portions.
B. The **"DATA-STATUS"** column indicates the status of the data in the “**TEXT**” column. The value “RAW-ORI” denotes that the entry is an original, unaltered response from the questionnaire. In contrast, values beginning with “DIS-” represent distortion-related sentences generated through back-translation techniques. For example, “DIS-EN” refers to data generated via English back-translation, while “DIS-WH” corresponds to back-translation from Chinese. Similar labels are used for Javanese ("DIS-JV"), Malay ("DIS-MS"), and Tagalog ("DIS-TG").
C. Each response was independently evaluated by two annotators, with their respective classifications recorded in the “**FIRST ANNOTATOR**” and “**SECOND ANNOTATOR**” columns. The dual-annotation approach enhances reliability, making this a valuable resource for natural language processing applications in mental health research. We obtained a Cohen’s Kappa coefficient of 1.00, which indicates perfect agreement between the two expert annotators. This value resulted from a complete match in their labeling decisions for each sentence in the dataset—both annotators assigned identical labels to all annotated texts as illustrated in that columns.

**Fig. 3:**
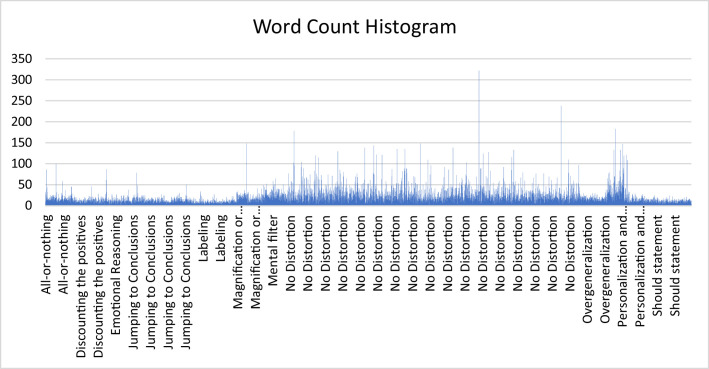
Word count histogram of all dataset.

Fig. 3 Shown “No Distortion” class dominates the dataset, both in quantity and in the variability of response length. The word counts in this class range widely, from very short to significantly long entries, reflecting the diverse nature of spontaneous, undistorted language responses provided by participants. In contrast, the distorted classes (e.g., All-or-nothing, Emotional Reasoning, Labeling, Magnification or Minimization) exhibit a more consistent and narrower word count distribution, with fewer instances overall. This pattern likely results from the augmentation process, where distorted instances were synthetically generated via back-translation, producing more uniform sentence lengths. Table 3 presents the detailed class distribution within the dataset that generated from original dataset and after back-translation process. While the overall split between distorted and non-distorted sentences shows reasonable balance (48.2% versus 51.8%), the distribution across specific cognitive distortion categories reveals notable imbalances. These variations in class frequency reflect natural differences in how frequently different types of cognitive distortions appear in spontaneous language. The dataset preserves original Indonesian language expressions while providing clear markers for distorted segments, enabling both broad classification and detailed analysis of specific distortion types.

**Table 3:** Dataset distribution of each class from original & after augmentation process.

Class ID	Distortion Class	Original Data Count	Post-Augmentation Data Count
0	No Distortion	2246	2246
1	Jumping to Conclusions	445	445
2	Labeling	390	390
3	Should statement	371	371
4	Discounting the positives	241	241
5	Mental filter	189	**200**
6	Personalization and Blame	284	284
7	Overgeneralization	215	215
8	All or nothing	158	**200**
9	Magnification or Minimization	85	**200**
10	Emotional Reasoning	38	**200**
	Total	4662	**4992**

## 4 Experimental Design, Materials and Methods

The dataset was collected through a structured questionnaire designed to elicit responses containing cognitive distortions. The questionnaire comprised 10 life-oriented questions targeting Indonesian respondents aged 18 years and older. These questions were formulated to elicit responses that reflect cognitive distortions, drawing upon established psychological theories and the professional experiences of the psychologists involved. The question design was developed in close collaboration with psychology experts to ensure alignment with recognized cognitive distortion typologies. As detailed in Table 4, each question was specifically tailored to target a distinct type of distortion in line with the meaning described in column “**Psychological Focus**”, thereby enabling systematic categorization during the annotation process. The questionnaire was distributed digitally, and responses were collected anonymously to preserve participant confidentiality. Responses were preprocessed to exclude non-informative entries (e.g., those under three words or containing only symbols) before annotation. The final curated dataset consisted of textual responses that were subsequently labeled by two experts, with distortion boundaries explicitly marked for analysis.

**Table 4:** Detail question (promt) in questionnaire.

Code	Question (Promt)	Expected Distortion Class	Psychological Focus
G1	Tell a story about an event in your life when you had the thought that you were the cause of a problem that occurred.	Personalization and Blame	Blaming yourself for a negative event that may not be entirely your responsibility.
G2	Tell a story about an event in your life when you had a thought that you only saw things in black and white or saw things in extreme success-failure/all-nothing.	All-or-nothing	Viewing situations in extremes, without recognizing intermediate possibilities.
G3	Tell a story about an event in your life when you had a thought that a negative event was the initial pattern of other negative events.	Overgeneralization	Interpreting a single negative event as a never-ending pattern of defeat or failure.
G4	Tell a story about an event in your life when you had a thought that was “must” or “should”.	Should Statement	Holding rigid rules about how you or others must behave, often leading to guilt or frustration.
G5	Tell a story about an event in your life when you had a thought that gave yourself or others negative labels.	Labeling	Assigning global negative traits to yourself or others based on specific behaviors.
G6	Tell a story about an event in your life when you had a thought that filtered negative information as a focus of your perspective.	Mental Filter	Focusing exclusively on negative details
G7	Tell a story about an event in your life when you had a thought that focused on negative information as a focus of your perspective (you rejected or ignored positive experiences you had).	Discounting the Positives	Dismissing positive experiences as if they don’t count or matter.
G8	Tell a story about an event in your life when you had a thought that exaggerated a problem and belittled/dwarfed the quality of your potential within yourself.	Magnification or Minimization	Exaggerating flaws or problems while minimizing your own strengths or achievements.
G9	Describe an event in your life when you came to a negative conclusion without any basis in fact.	Jumping to Conclusions	Making negative assumptions or predictions without sufficient evidence.
G10	Describe an event in your life when you had a thought that something was true because you felt it was true without considering logical reasons.	Emotional Reasoning	Believing something is true simply because it “feels” true, despite lack of logic or evidence.

**Fig. 4:**
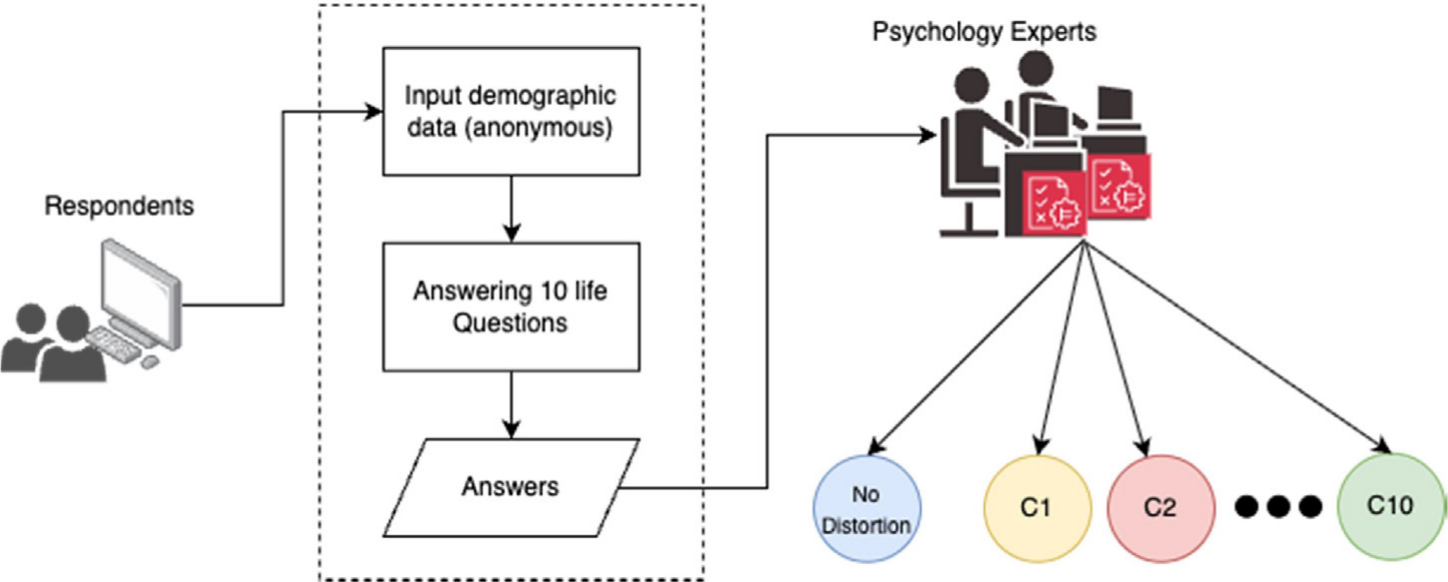
Data acquisition and annotation process chart.

The acquisition of this dataset uses an online questionnaire developed using Google Forms. Researchers distributed the survey link through random sampling to ensure diverse participation. The data collection and annotation process followed five sequential steps, as illustrated in Fig. 4.

1. First, respondents accessed the questionnaire electronically using various devices including computers, smartphones, or tablets.
2. Participants then provided demographic information while maintaining anonymity, disclosing only their initials and province of residence without sharing detailed personal identifiers.
3. Subsequently, respondents answered ten psychologically validated life questions designed to elicit potential cognitive distortions.
4. Following data collection, two expert annotators independently evaluated all responses using a standardized protocol. The annotation process involved: (1) identifying cognitive distortions based on established psychological theories and clinical experience, (2) classifying distortion types when present (C1-C10), and (3) marking distorted text segments by flanking them with “$” symbols. This symbol was selected purely for practical annotation purposes and subsequent NLP processing convenience, without theoretical significance.
5. Finally, the research team compiled the expert annotations, resolved any discrepancies through consensus, and stored the finalized dataset in *.csv format for accessibility and further analysis. The annotation methodology ensured systematic labeling while preserving the original linguistic expressions in the Indonesian language context.

In addition to the original sentence data, all augmented data were also reviewed and re-annotated by a psychologist to ensure compliance with the original annotation standards.

After obtaining all the required datasets, a baseline was established to evaluate the initial performance of the model in detecting and classifying 11 categories of cognitive distortions. During the distortion detection task, only the original dataset was used. In contrast, for the 11-class classification task, the dataset comprised all augmented distortion classes along with a randomly selected subset of 275 non-distortion instances, resulting in a total of 3,201 data points. The proportion of the dataset used for training is 80%, and the remaining is allocated for testing data. This applies to both detection and classification. This baseline was developed using four types of word embedding representations—BERT, fastText, Word2Vec, and GloVe-300d—combined with two classification algorithms: K-Nearest Neighbors (KNN) with K=9 and Support Vector Machine (SVM) using four distinct kernel types: linear, radial basis function (RBF), polynomial (POLY), and sigmoid. These combinations aim to identify the most effective initial configuration as a benchmark for the development of more advanced models in subsequent stages. As presented in Table 5, BERT paired with SVM (Linear) yielded the best performance in the binary detection task, demonstrating strong capability in distinguishing distorted from non-distorted sentences. In contrast, Word2Vec combined with SVM (RBF) produced the highest results in the 11-class classification task, effectively handling the finer-grained categorization of distortion types. The SVM model with the sigmoid kernel consistently yielded the poorest performance across both the binary detection and the 11-class classification tasks. This suggests that the sigmoid kernel may not be well-suited for capturing the complex patterns present in cognitive distortion texts within this dataset. However, these findings should be considered preliminary, as only a limited set of algorithmic combinations was explored, and comprehensive hyperparameter tuning has not yet been conducted.

**Table 5:** Baseline model performance.

Task	Model	Accuracy	Precision	Recall	F1-Score
Binary	KNN (K=9) + BERT	0.64	0.64	0.64	0.64
Binary	KNN (K=9) + fastText	0.64	0.67	0.65	0.63
Binary	KNN (K=9) + Word2Vec	0.65	0.71	0.66	0.63
Binary	KNN (K=9) + GloVe-300d	0.61	0.63	0.62	0.60
**Binary**	**SVM (Linear) + BERT**	**0.76**	**0.76**	**0.76**	**0.76**
Binary	SVM (RBF) + BERT	0.72	0.72	0.72	0.72
Binary	SVM (POLY) + BERT	0.72	0.72	0.72	0.72
Binary	SVM (Sigmoid) + BERT	0.70	0.70	0.70	0.70
Binary	SVM (Linear) + fastText	0.68	0.68	0.68	0.68
Binary	SVM (RBF) + fastText	0.74	0.75	0.75	0.74
Binary	SVM (POLY) + fastText	0.74	0.75	0.74	0.74
Binary	SVM (Sigmoid) + fastText	0.57	0.57	0.57	0.57
Binary	SVM (Linear) + Word2Vec	0.71	0.72	0.72	0.71
Binary	SVM (RBF) + Word2Vec	0.73	0.74	0.74	0.73
Binary	SVM (POLY) + Word2Vec	0.73	0.74	0.73	0.73
Binary	SVM (Sigmoid) + Word2Vec	0.57	0.57	0.57	0.57
Binary	SVM (Linear) + GloVe-300d	0.65	0.65	0.65	0.65
Binary	SVM (RBF) + GloVe-300d	0.68	0.68	0.68	0.68
Binary	SVM (POLY) + GloVe-300d	0.67	0.70	0.68	0.66
Binary	SVM (Sigmoid) + GloVe-300d	0.49	0.49	0.49	0.49
11 Class	KNN (K=9) + BERT	0.41	0.43	0.41	0.40
11 Class	KNN (K=9) + fastText	0.42	0.48	0.42	0.42
11 Class	KNN (K=9) + Word2Vec	0.42	0.53	0.42	0.43
11 Class	KNN (K=9) + GloVe-300d	0.32	0.35	0.32	0.33
11 Class	SVM (Linear) + BERT	0.58	0.58	0.58	0.58
11 Class	SVM (RBF) + BERT	0.46	0.48	0.46	0.45
11 Class	SVM (POLY) + BERT	0.49	0.49	0.49	0.48
11 Class	SVM (Sigmoid) + BERT	0.31	0.36	0.31	0.24
11 Class	SVM (Linear) + fastText	0.35	0.47	0.35	0.3
11 Class	SVM (RBF) + fastText	0.6	0.6	0.6	0.6
11 Class	SVM (POLY) + fastText	0.61	0.63	0.61	0.61
11 Class	SVM (Sigmoid) + fastText	0.47	0.52	0.47	0.46
11 Class	SVM (Linear) + Word2Vec	0.58	0.59	0.58	0.58
**11 Class**	**SVM (RBF) + Word2Vec**	**0.62**	**0.62**	**0.62**	**0.61**
11 Class	SVM (POLY) + Word2Vec	0.61	0.63	0.61	0.61
11 Class	SVM (Sigmoid) + Word2Vec	0.51	0.53	0.51	0.50
11 Class	SVM (Linear) + GloVe-300d	0.43	0.46	0.43	0.43
11 Class	SVM (RBF) + GloVe-300d	0.43	0.46	0.43	0.43
11 Class	SVM (POLY) + GloVe-300d	0.39	0.54	0.39	0.41
11 Class	SVM (Sigmoid) + GloVe-300d	0.25	0.31	0.25	0.24

## Limitations

While this dataset effectively supports automated detection of cognitive distortions due to its balanced distribution and the better result in baseline model evaluation, it presents challenges for fine-grained classification across all ten distortion categories. Although augmentation processes have been implemented, they are still not sufficient as the model has not performed optimally compared to the detection process. Various augmentation techniques need to be tried to obtain more relevant data, which can help improve the model's performance. The age distribution in this dataset is limited to individuals aged 18 to 30. Expanding the sample to include a broader age range would provide greater variation in sentence construction and expression styles, reflecting generational and temporal differences in life experiences.

## Ethics Statement

This research was approved by the Ethical Committee on Social Studies and Humanities- National Research and Innovation Agency of the Republic of Indonesia, reference number 966/KE.01/SK/12/2024. Informed consent was obtained from respondents while filling out the questionnaire on Google Forms.

## CRediT authorship contribution statement

**Nyoman Putra Sastra:** Methodology, Writing – review & editing, Supervision. **Linawati:** Methodology, Writing – review & editing, Supervision. **Gede Sukadarmika:** Methodology, Writing – review & editing, Supervision. **I.P.G.H. Suputra:** Conceptualization, Methodology, Software, Writing – original draft. **Ni Made Ariwilani:** Conceptualization, Data curation, Validation. **Ni Luh Indah Desira Swandi:** Data curation, Validation.

## Data Availability

Mendeley Data.Cognitive Distortion Dataset for Text Classification in Bahasa Indonesia (Original data)
